# Supplementary material for: Expression and Characterization of Two α-l-Arabinofuranosidases from Talaromyces amestolkiae: Role of These Enzymes in Biomass Valorization
Source: Int J Mol Sci. 2023 Jul 26;24(15):11997. doi: 10.3390/ijms241511997 (PMC10418624; doi:10.3390/ijms241511997)
Supplement: Supplementary file 1 [file ijms-24-11997-s001.zip › ijms-2509723-supplementary.pdf]

Supplementary Materials

GH62A

ATGATTCCTCTTGCTCCCGCTCTCCTTCTATTGTTACCGGTCGTCTCTGGCCTGGCACTTCCTACGGATG
AAACTGCACAAAACACCACAGAGGCAAGAGCCGGATGTGCTCTACCATCTAGCTATAGCTGGACGTCAAC
TGGTCCCCTGGCCAACCCAGCTTCTGGATTCCTCGCTTTGAAAGATTTCACCCACGTTCCTTACAACGGT
CAACATCTCGTATACGCGTCAGACGTCAACAATGGAGGAAACTACGGGTCTTTGAATTTTGCCCTCTTCT
CAGACTGGAACTCAATGGGCTCGGTGGCCCAACACACTATGAGTACTGGAGCTGTTGCTCCTCAACTGTT
CTTTTTTACTCCTAAGAACATCTGGGTGCTGGCATATGAATGGGGCGCAAGTCCTTTCAATTATAGGACC
TCCAGTGACCCCACCAATGCAAATGGATGGTCATCGCCTCAGTCACTCTACACTGGAAGTACTGGCTCTA
GCACGGGGCCTATTGACCCTTCTCTCATTGGCGATTCACAGAACATGTATCTCTTTTTTGCAGGCGATAA
TGGCAAGATATATCGAGCCAGCATGCCAATTGGTAATTTCCCGGGTAACTTTGGTTCTTCAGCACAAGTG
GTCCTCAGCGACTCGACCGTAAACCTCTTTGAGGCCGTCCAAGTGTACTCTGTCCAGGGCCAGAACCAGT
ATCTGATGATCGTGGAGGCAAGAGATCCCAGCGAAAACAGATACTTCCGTTCATTCACGGCCAGCAGTCT
GAGCGGTTCCTGGACACCTCAGGCCACCAGCGTGTCAGCACCGTTCGCGGGCAAGGCAAATAGCGGTGCC
AGCTGGACCAACGATATCAGCAGTGGTGATTTGATTAGAGCCAGTGCTGACCAGACCATGCCTATTGATG
CTTGCAATTTACAGCTCCTCTATCAGGGCAGAGCAACCAACTCTGGTGGCAGTTATAATGACTTGCCTTA
CCGTCCAGGTTTACTTACCCACAAATAG

MIPLAPALLLLLPVVSGLALPTDETAQNTTEARAGCALPSSYSWTSTGPLANPASGFLALKDFTHVPYNG
QHLVYASDVNNGGNYGSLNFALFSDWNSMGSVAQHTMSTGAVAPQLFFFTPKNIWVLAYEWGASPFNYRT
SSDPTNANGWSSPQSLYTGSTGSSTGPIDPSLIGDSQNMYLFFAGDNGKIYRASMPIGNFPGNFGSSAQV
VLSDSTVNLFEAVQVYSVQGQNQYLMIVEARDPSENRYFRSFTASSLSGSWTPQATSVSAPFAGKANSGA
SWTNDISSGDLIRASADQTMPIDACNLQLLYQGRATNSGGSYNDLPYRPGLLTHK

GH62B

ATGCTCTGGGTTGCGTCTTTGCTCCTGGTGATACCAGCTGTCACAGCTCAAGCATCGCTTTATGGTCAAT
GTAAGTCTCAATTTCCGAAGCATACTAGTCTTTATTTGCAGTAGAGCTAAGATCCTACGCAGGCGGTGGT
CAGGGCTGGACTGGAGCTACTACCTGTGTTTCTGGCTCTTGTTGTACCTACTCCAATGCCTGGTACTCGC
AGTGTCTTCCGTGCTCCGGCGGAAGTGGTACTACTACTACCTCAACTACTTTAACCACCACCACCAAGCC
AACCTCCACTTCCTCCACTACCTCCAGCTCTTCAAGCACATGCACTCTGCCGTCTCAGTATAAGTGGACT
TCAAGCGGATCGCTCGCGAACCCAGCGTCAGGCTATCTGTCGCTCAAGGACTTCACTCATGTGCCGTATA
ATGGCCAGCACCTTGTGTATGCTTCTGATGTTAATACCGGTGGAAGCTATGGTTCAATGAACTTTGCTCT
GTTCTCGAACTGGTCTCAAATGGCTTCCGCCACCAAGACGCAGATGAGCCAGGGAGCTGTTGCCCCTGAT
CTCCTGTACTTTGCACCAAAACAGATCTGGGTTCTAGGTTACGAATGGTGTGAATGGGCCTTTTGCTATC
TCACATCCACCAACCCTACCAATGCCAACGGCTGGTCTTCGCCACAACCGCTTTACCAAGGAACCTTATC
CGGAACAGGTCCTATTGATCCGGCAATTATCGCTGATGATACAAATGTTTATCTCTTCTTCGCCGGCGAC
AATGGCAATATCTACCGAGCTTCTATGCCCATTGGCAACTTCCCTGCTAGCTTTGGCTCTTCTGCTACAA
CCATCCTGACTGATTCGACATACAACCTCTTCGAAGCTGTCGAAGTCTACTCTATTCAGGGCCAGAACCT
TTACCTTATGATTGTTGAGTGCATTGGTTCCAACGGAAGATATTTCCGCTCCTTTACAGCTACGAGTCTA
GGTGGCACGTGGACTGCTCAAGCTAGCACTGAGTCAAACCCATTCGCTGGATATGCCAATAGCGGTGCTA
CCTGGACCAAGGATATCAGCAGTGGTGACCTTATCCGAAGCAGTGCTGATCAGACTAAACCAGTCGATCC
CTGCAACCTTCAGCTGCTCTACCAAGGAAGAAGCCCTAGCTCTGACGGCGATTCATATAACGATCTTCCT
TACCTCCCAGGTCTCTTGACCCTGTCCAAGTAG

MLWVASLLLVIPAVTAQASLYGQCGGQGWTGATTCVSGSCCTYSNAWYSQCLPCSGGSGTTTTSTTLTTT
TKPTSTSSTTSSSSSTCTLPSQYKWTSSGSLANPASGYLSLKDFTHVPYNGQHLVYASDVNTGGSYGSMN
FALFSNWSQMASATKTQMSQGAVAPDLLYFAPKQIWVLGYEWCEWAFCYLTSTNPTNANGWSSPQPLYQG
TLSGTGPIDPAIIADDTNVYLFFAGDNGNIYRASMPIGNFPASFGSSATTILTDSTYNLFEAVEVYSIQG
QNLYLMIVECIGSNGRYFRSFTATSLGGTWTAQASTESNPFAGYANSGATWTKDISSGDLIRSSADQTKP
VDPCNLQLLYQGRSPSSDGDSYNDLPYLPGLLTLSK

Figure S1. DNA and aminoacidic sequences of GH62A and GH62B. Signal peptide regions are indicated in red. The intron is highlighted in yellow.

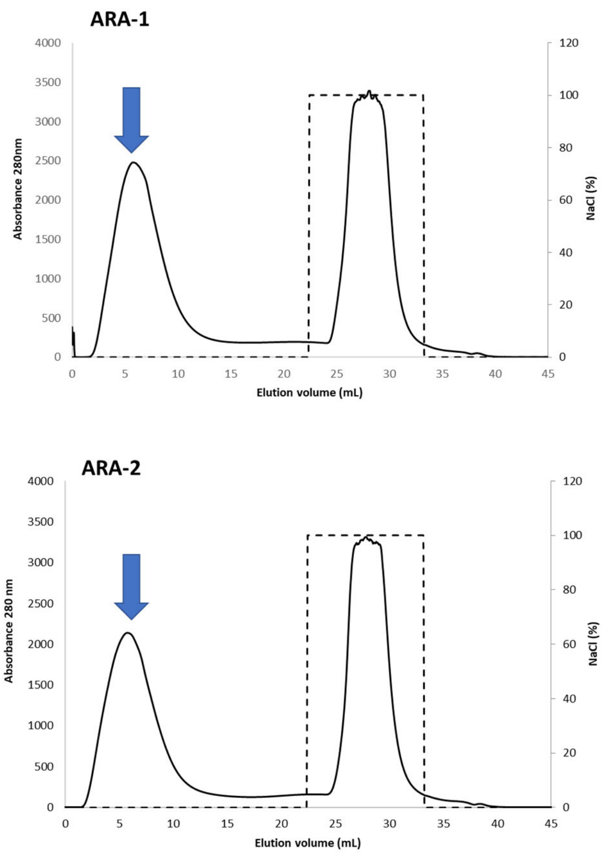

Figure S2. ARA-1 and ARA-2 purification using anion exchange chromatography. Dashed line, NaCl 1 M (%). Blue arrows point to peaks where enzymatic activity was detected.

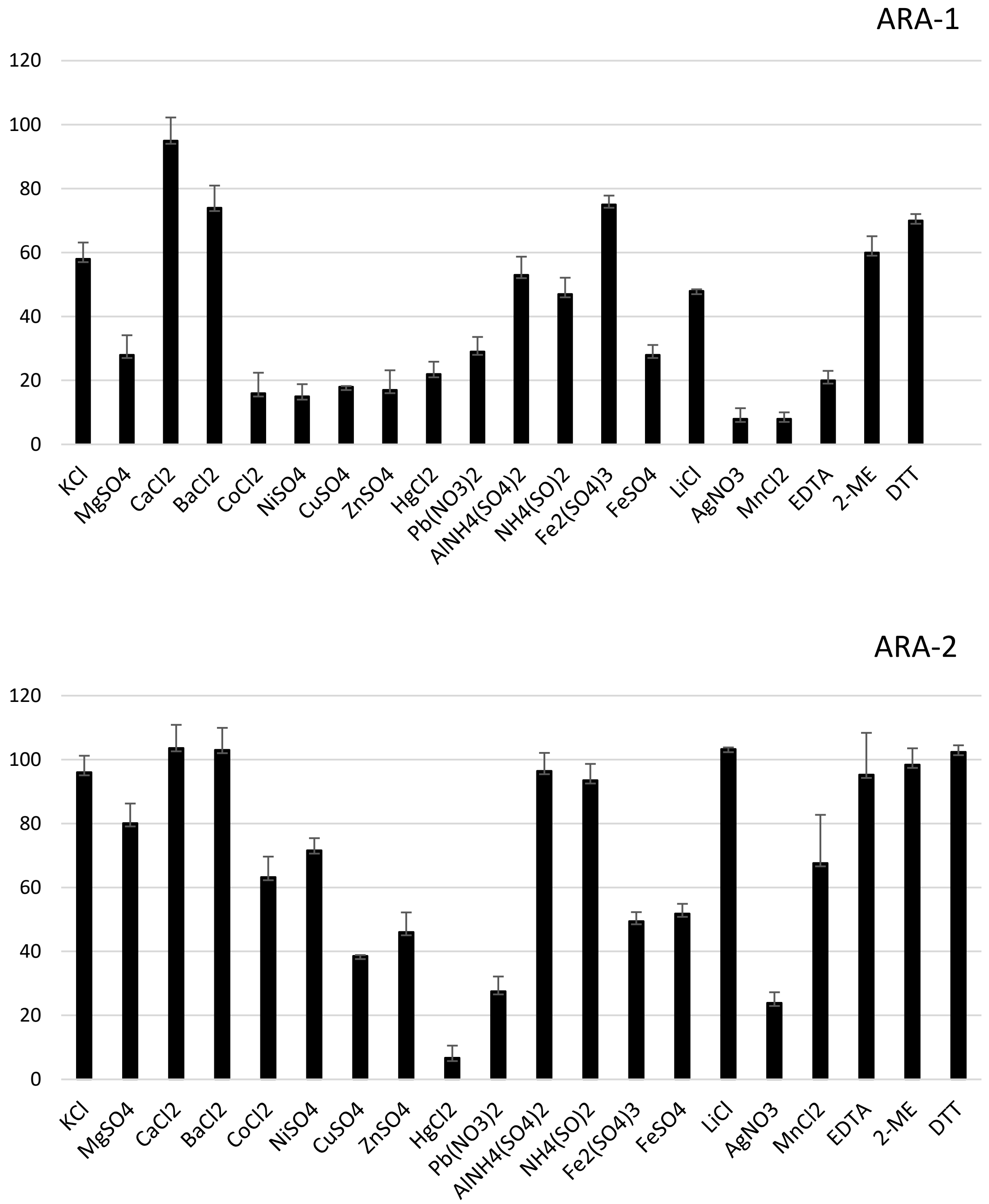

Figure S3. Effect of common compounds in ARA-1 and ARA-2 activity. Activity was measured under standard conditions and compared after incubation with the different chemicals. 100% activity was corresponded to the activity value determined without added compound.
